# Gene Expression Profiling in Peripheral Blood Mononuclear Cells of Patients with Common Variable Immunodeficiency: Modulation of Adaptive Immune Response following Intravenous Immunoglobulin Therapy

**DOI:** 10.1371/journal.pone.0097571

**Published:** 2014-05-15

**Authors:** Marzia Dolcino, Giuseppe Patuzzo, Alessandro Barbieri, Elisa Tinazzi, Monica Rizzi, Ruggero Beri, Giuseppe Argentino, Andrea Ottria, Claudio Lunardi, Antonio Puccetti

**Affiliations:** 1 Department of Immunology, Institute Giannina Gaslini, Genova, Italy; 2 Department of Medicine, University of Verona, Verona, Italy; 3 Department of Experimental Medicine, University of Genova, Genova, Italy; Cordelier Research Center, INSERMU872-Team16, France

## Abstract

**Background:**

Regular intravenous immunoglobulin treatment is used to replace antibody deficiency in primary immunodeficiency diseases; however the therapeutic effect seems to be related not only to antibody replacement but also to an active role in the modulation of the immune response. Common variable immunodeficiency is the most frequent primary immunodeficiency seen in clinical practice.

**Methods:**

We have studied the effect of intravenous immunoglobulin replacement in patients with common variable immunodeficiency by evaluating the gene-expression profiles from Affimetrix HG-U133A. Some of the gene array results were validated by real time RT-PCR and by the measurement of circulating cytokines and chemokines by ELISA. Moreover we performed FACS analysis of blood mononuclear cells from the patients enrolled in the study.

**Results:**

A series of genes involved in innate and acquired immune responses were markedly up- or down-modulated before therapy. Such genes included CD14, CD36, LEPR, IRF-5, RGS-1, CD38, TNFRSF25, IL-4, CXCR4, CCR3, IL-8. Most of these modulated genes showed an expression similar to that of normal controls after immunoglobulin replacement. Real time RT-PCR of selected genes and serum levels of IL-4, CXCR4 before and after therapy changed accordingly to gene array results. Interestingly, serum levels of IL-8 remained unchanged, as the corresponding gene, before and after treatment. FACS analysis showed a marked decrease of CD8+T cells and an increase of CD4+T cells following treatment. Moreover we observed a marked increase of CD23^−^CD27^−^IgM^−^IgG^−^ B cells (centrocytes).

**Conclusions:**

Our results are in accordance with previous reports and provide further support to the hypothesis that the benefits of intravenous immunoglobulin therapy are not only related to antibody replacement but also to its ability to modulate the immune response in common variable immunodeficiency.

## Introduction

Intravenous immunoglobulin (IVIg) is a therapeutic compound obtained from the serum IgG fraction pooled from several thousands of healthy donors [1], [2]. For many years IVIg has been used as a replacement therapy in a wide range of primary and secondary immunodeficiencies and now represents the first therapeutic option for antibody deficiencies [3]. IVIg is used in patients with X-linked agammaglobulinemia, X-linked hyper-IgM, severe combined immunodeficiency, Wiskott-Aldrich syndrome, selective IgG class deficiency and common variable immunodeficiency (CVID) [4]. CVID is the most common primary immunodeficiency seen in clinical practice. It represents a heterogeneous group of syndromes characterized by low serum levels of IgG, IgA and/or IgM, with decreased antibody production and impaired antibody response to both *polysaccharide* and *protein* antigens [5]. As a result of low antibody levels most patients have recurrent respiratory tract infections. The typical defect of CVID is the failure of B lymphocytes to differentiate into switched memory B cells and into plasma cells. Indeed reduced numbers of memory B cells have been associated with CVID, together with loss of isotype switched (CD27^+^IgD^−^IgM^−^) memory B cells [6].

Several abnormalities of T cells have also been described in CVID including oligoclonal expansion of CD8+T cells, and decreased numbers of CD4+T cells [7]. Moreover, T lymphocytes show an impaired secretion of several soluble mediators [8].

As far as the innate immune system concerns, some reports have shown that dendritic cells present a severely altered differentiation, maturation, function and reduced levels of costimulatory molecules that are critical for T cells stimulation [9]. Moreover a peripheral decreased number of natural killer cells [10] and monocytes alterations directly correlating with T cell activation markers and with B cell imbalances have been reported in CVID [11].

In CVID the importance of regular IVIg infusion has been initially attributed to the replacement of the missing antibodies and thereby to the prevention of recurrent infections [12]. More recently several lines of evidence suggest that the beneficial role of IVIg may not be limited to antibody replacement and that IVIg may also modulate the immune response [13], [14].

Aim of this study is to further clarify the effects of IVIg on the modulation of the immune response in patients with CVID.

## Methods

### Patients

A written informed consent was obtained from all the participants to the study. The study was approved by local Ethical Committee of the Azienda Ospedaliera Universitaria of Verona, Verona, Italy. All clinical investigation have been conducted according to the principles expressed in the Helsinky declaration.

We studied a cohort of 30 patients (10 males and 20 females, mean age: 44.8±12 years) affected by CVID, attending the Unit of Clinical Immunology at the University Hospital in Verona, Italy. All patients fulfilled the ESID/PAGID criteria for the diagnosis of CVID: marked decrease in IgG levels (at least 2 SD below the mean for patients' age) and marked decrease in at least one of the isotypes IgM or IgA, plus (a) onset of immunodeficiency after 2 years of age, (b) poor response to vaccines and (c) exclusion of other defined causes of hypogammaglobulinemia [5].

At enrolment, none of the patients had active infections or was affected by malignancies. Moreover, none was treated with antineoplastic or immunosuppressive drugs. All the patients were treated with regular monthly infusion of Immunoglobulins (Igs) at the dose of 0.4 g/kg. Twenty age- and sex- matched healthy donors served as controls.

### Preparation of cRNA and Array Analysis

Blood samples were collected in PAXgene Blood RNA tubes (PreAnalytiX, Hombrechtikon, Switzerland) and total RNA was extracted according to the protocol supplied by the manufacturer.

Preparation of cRNA hybridization and scanning of probe arrays for each samples were performed according to the protocols of the manufacturer (Affymetrix, Santa Clara, CA, USA) by Cogentech Affymetrix microarray unit (Campus IFOM IEO, Milan, Italy) using the Human Genome U133A 2.0 Gene Chip (Affymetrix). The Human Genome U133A Gene Chip is a single array representing 14,500 well-characterized human genes and including more than 22,000 probe sets and 500,000 distinct oligonucleotide features.

The different gene expression patterns were analyzed using the Gene Spring software, version 12.1 (Agilent Technologies, Santa Clara, CA, USA) that calculated a robust multi-array average of background-adjusted, normalized, and log-transformed intensity values applying the Robust Multi-Array Average algorithm (RMA).

With this software the mean optical background level for each array was subtracted from the signal intensity for each probe.

The normalized background-corrected data were transformed to the log_2_ scale. A signal log_2_ ratio of 1.0 indicates an increase of the transcript level by two-fold change (2 FC) and −1.0 indicates a decrease by two-fold change (−2 FC). A signal log_2_ ratio of zero would indicate no change. The unpaired t-test was performed to determine which genes were modulated at a significant level (*p*≤0.01) and *p* values were corrected for multiple testing by using Bonferroni correction. Finally, statistically significant genes were selected for final consideration when their expression was at least 2.0 fold different in the test sample versus control sample. Genes that passed both the *p*-value and the FC restriction were submitted to a functional classification according to the Gene Ontology (GO) annotations [15]–[17].

### Real Time RT-PCR

Total RNA was extracted from PBMC using TRIzol reagent (Invitrogen, Carlsbad, CA, USA), following manufacturer's instructions. First-strand cDNA was generated using the SuperScript III First-Strand Synthesis System for RT-PCR Kit (Invitrogen), with random hexamers, according to the manufacturer's protocol. RT product was aliquoted in equal volumes and stored at −20°C. PCR was performed in a total volume of 25 µl containing 1× Taqman Universal PCR Master mix, no AmpErase UNG and 2.5 µl of cDNA; pre-designed, Gene-specific primers and probe sets for each gene (RGS1 Hs01023772-m1) (IL8 Hs00174103-m1) (CCR3 Hs00266213-m1) (TNFRSF17 Hs 03045086-m1) were obtained from Assay-on-Demande Gene Expression Products (Applied Biosystems).

Real Time PCR reactions were carried out in a two-tube system and in singleplex. The Real Time amplifications included 10 minutes at 95°C (AmpliTaq Gold activation), followed by 40 cycles at 95°C for 15 seconds and at 60°C for one minute. Thermocycling and signal detection were performed with 7500 Sequence Detector (Applied Biosystems). Signals were detected according to the manufacturer's instructions. This technique allows the identification of the cycling point where PCR product is detectable by means of fluorescence emission (Threshold cycle or Ct value). As previously reported, the Ct value correlates to the starting quantity of target mRNA [18]. Relative expression levels were calculated for each sample after normalization against the housekeeping genes GAPDH, beta-actin and 18s ribosomal RNA (rRNA), using the ΔΔCt method for comparing relative fold expression differences [19], [20]. The data are expressed as fold change. Ct values for each reaction were determined using TaqMan SDS analysis software. For each amount of RNA tested triplicate Ct values were averaged. Because Ct values vary linearly with the logarithm of the amount of RNA, this average represents a geometric mean.

### Flow Cytometry Analysis

Blood samples were collected from patients before infusion of Igs and 7 days afterwards. To study T cell phenotype, a tube containing 2×10^6^ PBMCs in 100 µL of PBS was prepared. The same procedure was carried out to study the B cell phenotype.

T cells were stained with αCD3 PerCP, αCD4 APC-H7 and αCD8 FITC, while B cells were stained with αIgD FITC, IgM PE-Cy5, αCD23 PE, αCD27 APC, αCD5 PE-Cy7 and αCD19 APC-H7 antibodies. Staining was carried out at room temperature for 20 minutes. Cells were then washed in PBS at 1200 rpm for 5 minutes and resuspended in 400 µL of PBS. For each tube 20,000 events were acquired (CD3^+^ for T cell and CD19^+^ for B cell). All reagents were purchased from Becton Dickinson (San Jose, CA, USA), except for anti-IgD antibody (DAKO, Glostrup, Denmark). Samples were analysed on a FACSCanto II cytometer (Becton Dickinson) and data analysed by FlowJo 8.8.2 software (Tree Star, Ashland, OR, USA).

### ELISA assays

The detection of serum Interleukin-4 (IL-4) and IL-8 was performed using commercially available kits (R&D System, Inc., Minneapolis, MN, USA).

The kit for the detection of soluble chemokine (C-X-C motif) receptor 4 (CXCR4) was purchased from Cusabio (Wuhan, Hubei Province, P.R. China). All the kits were used according to the manufacturer's instructions.

## Results

### Gene array analysis

In order to evaluate the effects of IVIg, we first compared the gene expression profiles of PBMC obtained from 8 CVID patients before IVIg infusion with PBMC derived from 8 healthy donors.

When both a Bonferroni-corrected *P*-value criterion (p≤0.01) and a fold change criterion (FC≥2) were applied to the signal variation of every single gene to detect robust and statistically significant changes between baseline and experimental arrays [20], [21], 77 genes were differentially expressed in CVID patients, in particular 31 and 46 transcripts resulted, respectively, to be up- and down-regulated (Table S1).

Such transcripts were classified in functional categories according to GO annotations. Noteworthy the vast majority (75%) of modulated genes can be ascribed to gene categories related to innate (Table 1) and adaptive (Table 2) immune response.

**Table 1:** Genes related to adaptive immune responses in CVID patients before and after IVIg treatment.

Gene accession	Gene description	Gene Symbol	FC^a^	FC^b^
NM_000689	aldehyde dehydrogenase 1 family, member A1	ALDH1A1	3.47	3.14
NM_001837	chemokine (C-C motif) receptor 3	CCR3	7.40	5.12
NM_001774	CD37 molecule	CD37	2.35	2.31
NM_001775	CD38 molecule	CD38	−2.37	−2.80
NM_001828	Charcot-Leyden crystal protein	CLC	25.77	20.78
AF348491	chemokine (C-X-C motif) receptor 4	CXCR4	3.15	nc
AF064771	diacylglycerol kinase, alpha 80 kDa	DGKA	3.40	3.24
NM_004116	FK506 binding protein 1B, 12.6 kDa	FKBP1B	−2.10	nc
NM_001459	fms-related tyrosine kinase 3 ligand	FLT3LG	2.02	1.88
J03189	granzyme B	GZMB	−3.33	−4.27
U66825	major histocompatibility complex, class II, DR beta 1	HLA-DRB1	9.61	nc
NM_000589	interleukin 4	IL4	2.91	nc
NM_000584	interleukin 8	IL8	26.93	32.10
NM_001557	interleukin 8 receptor, beta	IL8RB	−2.89	−4.20
NM_002200	interferon regulatory factor 5	IRF5	3.27	nc
NM_002229	jun B proto-oncogene	JUNB	5.26	5.29
U50748	leptin receptor	LEPR	−2.51	nc
NM_021622	pleckstrin homology domain containing, family A member 1	PLEKHA1	−2.21	−2.43
NM_006404	protein C receptor, endothelial (EPCR)	PROCR	−2.34	−2.34
S69182	protein tyrosine phosphatase, non-receptor type 12	PTPN12	−2.11	nc
NM_002830	protein tyrosine phosphatase, non-receptor type 4	PTPN4	−2.48	−2.54
S59049	regulator of G-protein signaling 1	RGS1	11.31	nc
NM_013351	T-box 21	TBX21	−2.17	−2.51
X91817	transketolase-like 1	TKTL1	−4.54	−4.90
NM_001192	tumor necrosis factor receptor superfamily, member 17	TNFRSF17	−13.4	−12.8
NM_003790	tumor necrosis factor receptor superfamily, member 25	TNFRSF25	2.17	nc

a : before IVIG treatment.

b : after IVIG treatment.

nc: not significantly changed.

**Table 2:** Genes related to innate immune responses in CVID patients before and after IVIg treatment.

Gene accession	Gene description	Gene Symbol	FC^a^	FC^b^
J04132	CD247 molecule	CD247	−2.23	−2.48
NM_000591	CD14 molecule	CD14	2.04	nc
Z25431	NIMA (never in mitosis gene a)-related kinase 1	NEK1	−2.01	nc
NM_014840	NUAK family, SNF1-like kinase, 1	NUAK1	−3.07	nc
D38122	Fas ligand (TNF superfamily, member 6)	FASLG	−4.81	−5.75
U26455	ataxia telangiectasia mutated	ATM	−2.21	nc
NM_002048	growth arrest-specific 1	GAS1	−4.93	nc
NM_005433	v-yes-1 Yamaguchi sarcoma viral oncogene homolog 1	YES1	−2.60	−2.54
NM_001062	transcobalamin I (vitamin B12 binding protein, R binder family)	TCN1	6.31	5.88
J04162	Fc fragment of IgG, low affinity IIIa, receptor (CD16a)	FCGR3A	−2.60	−3.34
NM_000139	membrane-spanning 4-domains, subfamily A, member 2	MS4A2	4.31	1.92
NM_000878	interleukin 2 receptor, beta	IL2RB	−2.23	−2.67
NM_000305	paraoxonase 2	PON2	−2.16	−2.21
NM_004417	dual specificity phosphatase 1	DUSP1	3.54	3.14
NM_001150	alanyl (membrane) aminopeptidase	ANPEP	5.62	5.61
NM_004334	bone marrow stromal cell antigen 1	BST1	2.00	2.11
NM_013252	C-type lectin domain family 5, member A	CLEC5A	2.65	nc
NM_000760	colony stimulating factor 3 receptor (granulocyte)	CSF3R	2.78	2.54
NM_024636	STEAP family member 4	STEAP4	2.02	2.17
NM_002438	mannose receptor, C type 1	MRC1	−2.46	−2.16
NM_004829	natural cytotoxicity triggering receptor 1	NCR1	−2.33	nc
NM_006291	tumor necrosis factor, alpha-induced protein 2	TNFAIP2	2.32	nc
NM_001953	thymidine phosphorylase	TYMP	2.13	1.89
NM_000860	hydroxyprostaglandin dehydrogenase 15-(NAD)	HPGD	−2.63	−2.47
NM_003407	zinc finger protein 36, C3H type, homolog	ZFP36	4.45	nc
NM_000072	CD36 molecule (thrombospondin receptor)	CD36	2.12	nc
NM_000271	Niemann-Pick disease, type C1	NPC1	−2.32	−2.55
NM_002112	histidine decarboxylase	HDC	6.50	5.75
AF065214	phospholipase A2, group IVC (cytosolic, calcium-independent)	PLA2G4C	−2.81	−3.02
NM_004163	RAB27B, member RAS oncogene family	RAB27B	−2.37	−2.58
NM_001553	insulin-like growth factor binding protein 7	IGFBP7	−3.09	−3.19
NM_005764	PDZK1 interacting protein 1	PDZK1IP1	−6.60	−6.74

a : before IVIG treatment.

b : after IVIG treatment.

nc: not significantly changed.

Such genes included CD14 molecule (CD14, FC 2), leptin receptor (LEPR, FC −2.5), CD38 molecule (CD38, FC −2.4), RGS1, (FC 11.3), TNFRSF25 (FC 2.2), interleukin 4 (IL4, FC 2.9), CXCR4 (FC 3.1), CCR3, (FC 7.4), IL8 (FC 26.9).

**Figure 1:**
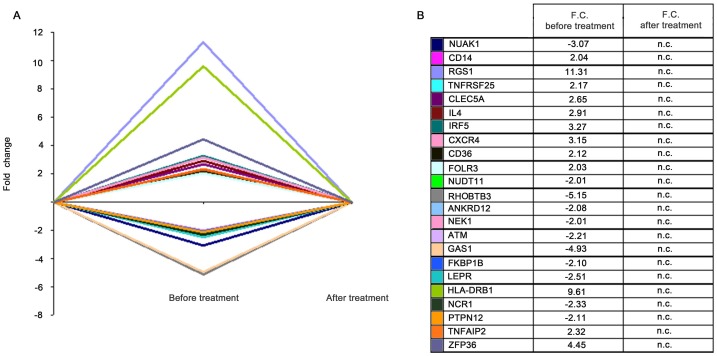
Genes modulated by IVIg infusion. A. Graphical representation of the expression of the 23 genes returned to the levels of normal controls after IVIg infusion. B. FC expression, before and after therapy, of the genes represented in panel A with the corresponding colours.

In order to evaluate the potential effect of IVIg infusion on the immune response in CVID patients, we analysed the transcriptional profiles obtained from the same CVID patients 3 days after IVIg infusion. When we compared the gene expression levels before and after treatment we observed that 23 of the 77 genes returned to baseline expression levels showing an expression similar to that of normal controls (Figure 1).

We then analysed genes modulated only after IVIg treatment using the same criteria described above. Thirty-five genes were differentially expressed in CVID patients after IVIg infusion, in particular 9 and 26 transcripts resulted, respectively, to be up- and down-regulated (Table S2). Before treatment such genes did not show any significant variation in their FC when compared to healthy subjects.

Noteworthy most of the modulated genes (27 out of 35, 77%) belong to functional categories related to adaptive immune response, including chemokine (C-X-C motif) ligand 2 (CXCL2; FC 12.6), C-type lecin domain family 4 member E (CLEC4E; FC 2.1), CD226 (FC −3.1), Interleukin 16 (IL16; FC −2.3).

**Figure 2:**
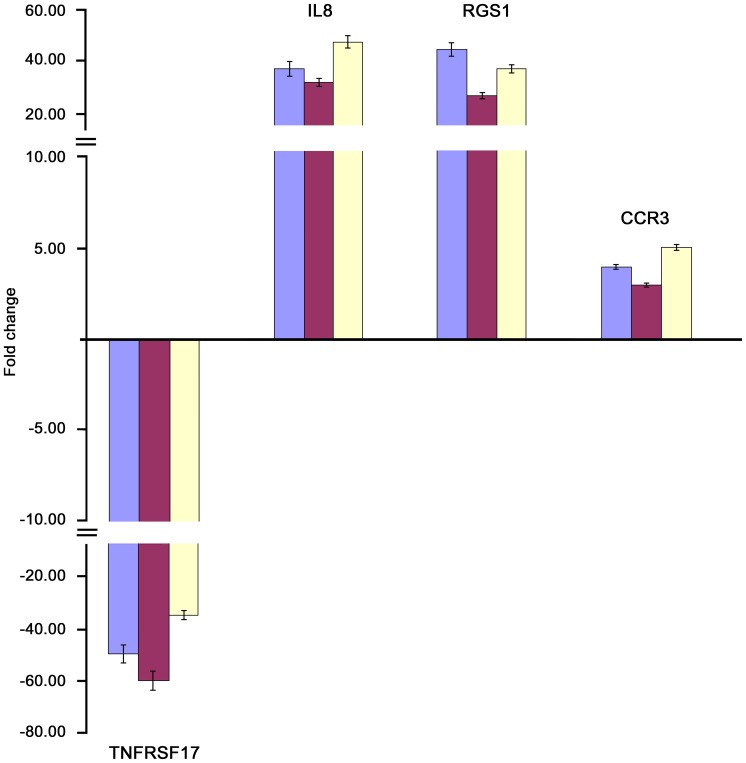
Real time RT-PCR of some modulated genes confirms the results of gene array analysis. Genes selected for validation in PBMC of CVID patients before IVIg treatment. IL8, RGS1 and CCR3 transcripts were increased, whereas TNFRSF17 transcript was decreased in CVID patients when compared to healthy donors. Relative expression levels were calculated for each sample after normalization against the housekeeping genes 18s rRNA, beta-actin and GAPDH. Experiments have been conducted in triplicates. Housekeeping genes: blue bar  = 18s rRNA; magenta bar: beta-actin; yellow bar: GAPDH.

Some of the genes modulated at the gene array analysis were validated by real time RT-PCR (Figure 2).

All together the results so far obtained suggest that IVIg infusion has a profound impact on transcriptional profiles of PBMC of CVID patients, indicating a possible immunomodulatory effect of IVIg therapy.

### FACS Analysis

We next wanted to verify whether the transcriptional profiles modulated by IVIg were paralleled by phenotypic modification of the T and B cell subsets in CVID patients. To this aim we performed a FACS analysis of PBMC collected from the patients immediately before and a week after IVIg infusion.

**Figure 3:**
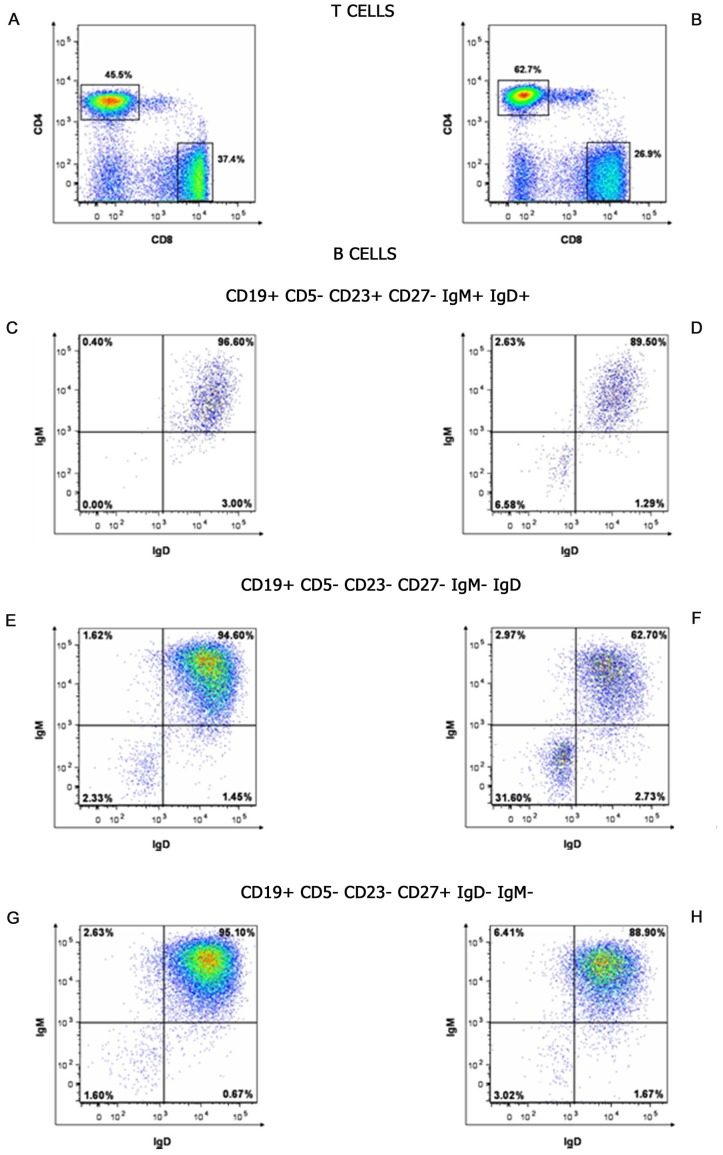
Flow cytometric analysis of T and B cell populations in CVID patients before and after IVIg infusion. Data are representative of all the subjects studied. A and B panels show the percentages of CD4+ and CD8+T cell populations before and after IVIg infusion, respectively. Percentages of naïve B cells, centrocytes and switched memory B cells before and after IVIg infusion are displayed in C vs D, E vs F and G vs H panels, respectively.

When we studied the T cell subsets, we found that the percentage of CD4+T cells increased following IVIg infusion in all 30 patients enrolled in the study. The difference observed before and after the treatment ranged between 11% and 17.2%. The mean value +/− SEM of CD4+T cells was 39.18±3.27 before and 51.8±4.42 after treatment. Moreover, in all the 30 patients studied, the percentage of CD8+T cells decreased with a range between 5.3% and 11.1% following Ig replacement therapy (Figure 3 A, B). Indeed the mean value of CD8+T cells varied from 45.28±3.3 before therapy to 38.43±4.6 after IVIg therapy.

We then focused our attention on the B cell repertoire by analysing three cell subpopulations at different stages of B cell differentiation. In particular we considered: naïve B cells (CD19^+^CD5^−^CD23^+^CD27^−^IgM^+^IgD^+^), centrocytes (CD19^+^CD5^−^CD23^−^CD27^−^IgM^−^IgD^−^) and switched memory B cells (CD19^+^CD5^−^CD23^−^CD27^+^IgM^−^IgD^−^). The IVIg treatment induced the following modifications in these cell subsets:

- the percentage of CD23^+^CD27^−^IgM^+^IgD^+^ B cells decreased with a range between 2.4% and 7.1%. The mean values varied from 93.45±2.23 to 89.6±0.07.
- the percentage of CD23^−^CD27^−^IgM^−^IgD^−^ B cells increased in all the patients and the difference ranged between 10.1% and 29.3%. The mean values varied from 2.6±0.19 to 17.71±9.83.
- finally the percentage of CD23^−^CD27^+^IgM^−^IgD^−^ B cells increased in all the patients between 0.4% and 1.4%. The mean values varied from 3.02±1 to 3.94±0.65 (Figure 3 C, D, E, F, G, H).

Taken together, these results indicate that IVIg treatment has an active role in modulating the adaptive immune response in CVID.

### Detection of soluble mediators in CVID sera

**Figure 4:**
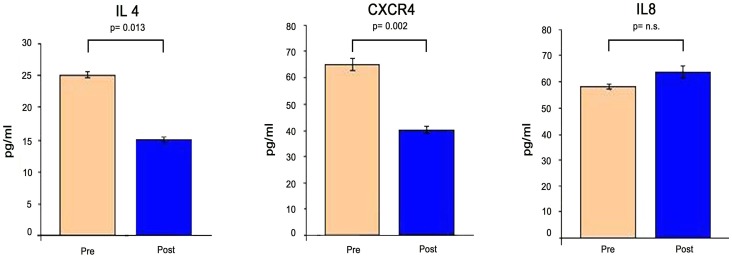
Serum levels of selected soluble mediators in CVID patients before and after IVIg infusion. The histogram represents the mean of the results obtained in 30 patients. Results are expressed in pg/ml. *p* values calculated using the Student's t-test for paired samples.

The analysis of gene expression profiles were paralleled by the detection of some of the corresponding soluble mediators in the sera of patients with CVID before and after Igs replacement therapy. We decided to analyse the levels of IL-4, CXCR4 and IL-8 (Figure 4). IL-4 is a pleiotropic cytokine produced by Th2 cells. CXCR4 is an alpha-chemokine receptor with a potent chemotactic activity for lymphocytes [22]. IL-8 is a chemokine produced by macrophages which induces chemotaxis and phagocytosis. Figure 4 shows the concentration of these molecules in the sera of CVID patients before and 72 hours after IVIg therapy. Both IL-4 and CXCR4 decreased after Ig infusion in agreement with the gene array analysis [23], [24]. On the contrary IL-8 level was not significantly modified after Ig treatment as it happened for the expression of the coding gene [25]. These data suggest that gene expression is paralleled by secretion of the corresponding molecules in the sera of CVID patients.

## Discussion

The results we report here on the ability of IVIg therapy to modulate the immune response in CVID patients are in agreement with previous findings and support the hypothesis the IVIg treatment has many effects, also at gene expression level, apart from the antibody replacement.

Indeed IVIg is worldwide used as replacement therapy in primary immunodeficiency because of its ability to reduce the frequency and severity of infections and therefore to increase life expectancy in immune compromised patients. Despite the evident efficacy of IVIg therapy, little is known on its effects apart from antibody replacement.

In the last few years much effort has been made in order to better understand the underlying mechanisms of the beneficial effects of IVIg therapy, hypothesizing a possible role of IVIg in the modulation of the immune response. Indeed the group of Kaveri has reported that IVIg induces proliferation of B lymphocytes and immunoglobulin synthesis in CVID patients, apparently rectifying the defective signalling of B cells, normally provided by T cells [26]. Moreover Paquin-Proulx *et al* observed that in patients with CVID there was a partial restore of CD4+/CD8+T cell ratio together with the reduction of CD8+T cells activation after IVIg infusion [27].

Our work aimed at clarifying some of these aspects, in particular we focused our attention on the potential effects of IVIg on cells of the adaptive immune response. For this purpose we used, for the first time, a gene array approach, which provides a global vision of the effects of IVIg therapy on gene transcription.

Our data show that Igs treatment has a profound impact on gene expression with a selective modulation of gene clusters involved in innate and adaptive immunity. We then evaluated some features of adaptive immunity since the CVID is known to affect mainly this branch of the immune system [4]–[6]. The changes observed in gene transcription were paralleled by important phenotypic modifications in T and B cell subsets percentage in all the patients enrolled in the study. Such modifications include the expansion of CD4+ helper T cells and the reduction of CD8+ cytotoxic T cells. This effect on CD8+T cells is in accordance with recent findings in animal models [28], [29]; indeed IVIg treatment has been reported to decrease in *vitro* response of antigen specific CD8+T cells, suggesting a similar mechanism in *vivo* in patients with inflammatory and autoimmune diseases, characterized by self-reactive cytotoxicity [30].

These phenotypic modifications are in agreement with the gene expression data. For example the expression of the TNFRSF25 gene, known to promote CD8+T cells survival [31], is up-regulated before IVIg replacement and returns to baseline levels after the infusion, in accordance with the reduction of CD8+T cells. On the other hand the LEPR gene which plays an important role in the proliferation of CD4+T cells [32], [33], is down-regulated before IVIg and its expression is similar to that of healthy controls after the treatment. The modulation of these two genes may therefore be related to the increase of CD4+ cells and the decrease of CD8+ cells after IVIg.

Similarly, transcriptional changes of genes involved in B cell maturation may lead to the modifications of the B cell phenotype induced by IVIg. In this regard it is important to point out the role of CD38 and RGS1 gene in the progression of B cells from the early stages to switched memory B cells [34]–[36].

Even more interestingly, IVIg induced an increase in centrocytes and switched memory B cells with a reduction of naïve B cells. We have recently proposed that the defective B cells maturation in CVID patients may lay in the passage between centroblastasts and centrocytes. Therefore the increase in centrocytes is a surprising and unexpected effect of IVIg [37].

Some of the gene array data were also confirmed by detecting serum levels of soluble mediators such as IL4, IL8 and CXCR4. The increased expression of genes encoding for IL-4 and CXCR4 before IVIg infusion and the normalization of gene expression together with the decrease in the circulant cytokine and chemokine may indicate that there is a dysregulation also of T helper subsets and of neutrophils in patients affected by CVID. Moreover it has been shown that increased expression of CXCR4 modulates the expression of pro- and anti-apoptotic molecules such as Bad, Bax and Bcl-2. Finally in mice knocked out for CXCR4 a defective B cell maturation has been observed. Therefore the increased expression of CXCR4 in patients with CVID may represent a possible attempt to induce B cell maturation [38], [39].

Our results further support the suggested immunomodulatory properties of IVIg therapy; indeed we consider particularly interesting the ability of this therapy to induce gene modulation, as shown by the results of the gene array analysis. A precise evaluation of the mechanisms underlying these phenomena is beyond the scope of the present work. However several hypotheses have been proposed to explain the mechanisms of action of Igs and both Fc dependent and Fab dependent mechanisms have been implicated in the potential immunomodulatory action of IVIg [13], [14]. In the last few years the use of IVIg has been extended to a selected group of autoimmune and inflammatory diseases such as Kawasaki disease, steroid-resistant or aggressive dermatomyositis, chronic inflammatory demyelinating polyneuropathy. Our observations on the immunomodulatory properties of IVIg may partially explain the beneficial effects of this therapy in autoimmune and inflammatory diseases where Igs replacement is not required.

In conclusion our work is in accordance with previous reports and provide further demonstration of the immunomodulatory action of IVIg therapy in CVID. The next steps of the research need to clarify both the detailed mechanisms of this immunomodulation and whether similar or some other immunomodulatory effects are present following IVIg infusion used in autoimmune and inflammatory diseases.

The results of our research may also have an important role in clinical practice, suggesting a different timing in Igs infusion: this is a fundamental aspect, considering the problems related to the shortage of Igs supply.

## Supporting Information

Table S1 **Gene expression in CVID before and after IVIG treatment.**
(DOC)

Table S2 **Annotated genes differentially expressed in CVID only after IVIG treatment.**
(DOC)
