# Supplementary material for: Gene Expression Profiling in Peripheral Blood Mononuclear Cells of Patients with Common Variable Immunodeficiency: Modulation of Adaptive Immune Response following Intravenous Immunoglobulin Therapy
Source: PLoS One. 2014 May 15;9(5):e97571. doi: 10.1371/journal.pone.0097571 (PMC4022614; doi:10.1371/journal.pone.0097571)
Supplement: Table S1 — Gene expression in CVID before and after IVIG treatment. (DOC) [file pone.0097571.s001.doc]

| **Table S1. Gene expression in CVID before and after IVIG treatment.** | | | | | | | |
| --- | --- | --- | --- | --- | --- | --- | --- |
| **Probe Set ID** | **Gene Symbol** | **Gene Title** | **FC^a^** | ***p* value^a^** | **FC^b^** | ***p* value^b^** | Accession number |
| |  |  |  |  |  | | --- | --- | --- | --- | --- |   210031_at | CD247 | CD247 molecule | -2.23 | 0.002 | -2.48 | 0.000 | J04132 |
| 201743_at | CD14 | CD14 molecule | 2.04 | 0.004 | nc | 0.011 | NM_000591 |
| 211086_x_at | NEK1 | NIMA (never in mitosis gene a)-related kinase 1 | -2.01 | 0.000 | nc | 0.012 | Z25431 |
| 204589_at | NUAK1 | NUAK family, SNF1-like kinase, 1 | -3.07 | 0.005 | nc | 0.013 | NM_014840 |
| 210865_at | FASLG | Fas ligand (TNF superfamily, member 6) | -4.81 | 0.001 | -5.75 | 0.003 | D38122 |
| 210858_x_at | ATM | ataxia telangiectasia mutated | -2.21 | 0.006 | nc | 0.012 | U26455 |
| 204457_s_at | GAS1 | growth arrest-specific 1 | -4.93 | 0.003 | nc | 0.011 | NM_002048 |
| 202976_s_at | RHOBTB3 | Rho-related BTB domain containing 3 | -5.15 | 0.007 | nc | 0.005 | NM_014899 |
| 202933_s_at | YES1 | v-yes-1 Yamaguchi sarcoma viral oncogene homolog 1 | -2.60 | 0.001 | -2.54 | 0.000 | NM_005433 |
| 219024_at | PLEKHA1 | pleckstrin homology domain containing, family A (phosphoinositide binding specific) member 1 | -2.21 | 0.000 | -2.43 | 0.002 | NM_021622 |
| 210164_at | GZMB | granzyme B (granzyme 2, cytotoxic T-lymphocyte-associated serine esterase 1) | -3.33 | 0.002 | -4.27 | 0.001 | J03189 |
| 204192_at | CD37 | CD37 molecule | 2.35 | 0.004 | 2.31 | 0.012 | NM_001774 |
| 211272_s_at | DGKA | diacylglycerol kinase, alpha 80kDa | 3.40 | 0.011 | 3.24 | 0.015 | AF064771 |
| 206857_s_at | FKBP1B | FK506 binding protein 1B, 12.6 kDa | -2.10 | 0.001 | nc | 0.013 | NM_004116 |
| 205513_at | TCN1 | transcobalamin I (vitamin B12 binding protein, R binder family) | 6.31 | 0.013 | 5.88 | 0.010 | NM_001062 |
| 216834_at | RGS1 | regulator of G-protein signaling 1 | 11.31 | 0.015 | nc | 0.003 | S59049 |
| 205692_s_at | CD38 | CD38 molecule | -2.37 | 0.002 | -2.80 | 0.002 | NM_001775 |
| 206641_at | TNFRSF17 | tumor necrosis factor receptor superfamily, member 17 | -13.40 | 0.000 | -12.79 | 0.003 | NM_001192 |
| 204006_s_at | FCGR3A | Fc fragment of IgG, low affinity IIIa, receptor (CD16a) | -2.60 | 0.001 | -3.34 | 0.011 | J04162 |
| 209894_at | LEPR | leptin receptor | -2.51 | 0.000 | nc | 0.012 | U50748 |
| 219423_x_at | TNFRSF25 | tumor necrosis factor receptor superfamily, member 25 | 2.17 | 0.012 | nc | 0.011 | NM_003790 |
| 221491_x_at | HLA-DRB1 | major histocompatibility complex, class II, DR beta 1 | 9.61 | 0.000 | nc | 0.001 | U66825 |
| 207496_at | MS4A2 | membrane-spanning 4-domains, subfamily A, member 2 | 4.31 | 0.015 | 1.92 | 0.003 | NM_000139 |
| 205291_at | IL2RB | interleukin 2 receptor, beta | -2.23 | 0.008 | -2.67 | 0.002 | NM_000878 |
| 207008_at | IL8RB | interleukin 8 receptor, beta | -2.89 | 0.015 | -4.20 | 0.010 | NM_001557 |
| 201876_at | PON2 | paraoxonase 2 | -2.16 | 0.001 | -2.21 | 0.005 | NM_000305 |
| 201041_s_at | DUSP1 | dual specificity phosphatase 1 | 3.54 | 0.002 | 3.14 | 0.001 | NM_004417 |
| 202888_s_at | ANPEP | alanyl (membrane) aminopeptidase | 5.62 | 0.000 | 5.61 | 0.000 | NM_001150 |
| 205715_at | BST1 | bone marrow stromal cell antigen 1 | 2.00 | 0.011 | 2.11 | 0.010 | NM_004334 |
| 219890_at | CLEC5A | C-type lectin domain family 5, member A | 2.65 | 0.013 | nc | 0.003 | NM_013252 |
| 203591_s_at | CSF3R | colony stimulating factor 3 receptor (granulocyte) | 2.78 | 0.009 | 2.54 | 0.013 | NM_000760 |
| 220187_at | STEAP4 | STEAP family member 4 | 2.02 | 0.012 | 2.17 | 0.012 | NM_024636 |
| 204438_at | MRC1 | mannose receptor, C type 1 | -2.46 | 0.005 | -2.16 | 0.012 | NM_002438 |
| 207860_at | NCR1 | natural cytotoxicity triggering receptor 1 | -2.33 | 0.009 | nc | 0.011 | NM_004829 |
| 205171_at | PTPN4 | protein tyrosine phosphatase, non-receptor type 4 (megakaryocyte) | -2.48 | 0.003 | -2.54 | 0.001 | NM_002830 |
| 216915_s_at | PTPN12 | protein tyrosine phosphatase, non-receptor type 12 | -2.11 | 0.000 | nc | 0.011 | S69182 |
| 220684_at | TBX21 | T-box 21 | -2.17 | 0.006 | -2.51 | 0.002 | NM_013351 |
| 206980_s_at | FLT3LG | fms-related tyrosine kinase 3 ligand | 2.02 | 0.001 | 1.88 | 0.011 | NM_001459 |
| 207539_s_at | IL4 | interleukin 4 | 2.91 | 0.012 | nc | 0.013 | NM_000589 |
| 202510_s_at | TNFAIP2 | tumor necrosis factor, alpha-induced protein 2 | 2.32 | 0.007 | nc | 0.012 | NM_006291 |
| 203650_at | PROCR | protein C receptor, endothelial (EPCR) | -2.34 | 0.010 | -2.34 | 0.000 | NM_006404 |
| 204858_s_at | TYMP | thymidine phosphorylase | 2.13 | 0.010 | 1.89 | 0.013 | NM_001953 |
| 202859_x_at | IL8 | interleukin 8 | 26.93 | 0.014 | 32.10 | 0.006 | NM_000584 |
| 206207_at | CLC | Charcot-Leyden crystal protein | 25.77 | 0.000 | 20.78 | 0.000 | NM_001828 |
| 208304_at | CCR3 | chemokine (C-C motif) receptor 3 | 7.40 | 0.005 | 5.12 | 0.007 | NM_001837 |
| 211919_s_at | CXCR4 | chemokine (C-X-C motif) receptor 4 | 3.15 | 0.013 | nc | 0.013 | AF348491 |
| 203914_x_at | HPGD | hydroxyprostaglandin dehydrogenase 15-(NAD) | -2.63 | 0.007 | -2.47 | 0.013 | NM_000860 |
| 201531_at | ZFP36 | zinc finger protein 36, C3H type, homolog (mouse) | 4.45 | 0.014 | nc | 0.011 | NM_003407 |
| 206488_s_at | CD36 | CD36 molecule (thrombospondin receptor) | 2.12 | 0.008 | nc | 0.013 | NM_000072 |
| 204160_s_at | ENPP4 | ectonucleotide pyrophosphatase/phosphodiesterase 4 (putative function) | -3.10 | 0.008 | -3.64 | 0.003 | NM_014936 |
| 203060_s_at | PAPSS2 | 3'-phosphoadenosine 5'-phosphosulfate synthase 2 | -3.09 | 0.004 | -2.64 | 0.002 | NM_004670 |
| 202679_at | NPC1 | Niemann-Pick disease, type C1 | -2.32 | 0.006 | -2.55 | 0.005 | NM_000271 |
| 214183_s_at | TKTL1 | transketolase-like 1 | -4.54 | 0.011 | -4.90 | 0.006 | X91817 |
| 219821_s_at | GFOD1 | glucose-fructose oxidoreductase domain containing 1 | -3.04 | 0.002 | -3.01 | 0.006 | NM_018988 |
| 204811_s_at | CACNA2D2 | calcium channel, voltage-dependent, alpha 2/delta subunit 2 | -2.59 | 0.005 | -2.97 | 0.001 | NM_006030 |
| 204836_at | GLDC | glycine dehydrogenase (decarboxylating) | -2.58 | 0.001 | -2.72 | 0.002 | NM_000170 |
| 202218_s_at | FADS2 | fatty acid desaturase 2 | -2.19 | 0.000 | -1.79 | 0.002 | NM_004265 |
| 214474_at | PRKAB2 | protein kinase, AMP-activated, beta 2 non-catalytic subunit | -2.17 | 0.002 | -2.08 | 0.000 | NM_005399 |
| 204718_at | EPHB6 | EPH receptor B6 | 2.16 | 0.015 | 2.05 | 0.004 | NM_004445 |
| 204256_at | ELOVL6 | ELOVL family member 6, elongation of long chain fatty acids (FEN1/Elo2, SUR4/Elo3-like, yeast) | -4.10 | 0.013 | -5.22 | 0.009 | NM_024090 |
| 206371_at | FOLR3 | folate receptor 3 (gamma)* | 2.03 | 0.009 | nc | 0.007 | NM_000804 |
| 219855_at | NUDT11 | nudix (nucleoside diphosphate linked moiety X)-type motif 11 | -2.01 | 0.007 | nc | 0.005 | NM_018159 |
| 203211_s_at | MTMR2 | myotubularin related protein 2 | -2.26 | 0.002 | -2.19 | 0.001 | NM_016156 |
| 206233_at | B4GALT6 | UDP-Gal:betaGlcNAc beta 1,4- galactosyltransferase, polypeptide 6 | -3.02 | 0.008 | -3.80 | 0.010 | AF097159 |
| 207067_s_at | HDC | histidine decarboxylase | 6.50 | 0.002 | 5.75 | 0.001 | NM_002112 |
| 207357_s_at | GALNT10 | UDP-N-acetyl-alpha-D-galactosamine:polypeptide N-acetylgalactosaminyltransferase 10 (GalNAc-T10) | -2.02 | 0.003 | -1.95 | 0.008 | NM_017540 |
| 204293_at | SGSH | N-sulfoglucosamine sulfohydrolase | 2.20 | 0.007 | 2.19 | 0.002 | NM_000199 |
| 212224_at | ALDH1A1 | aldehyde dehydrogenase 1 family, member A1 | 3.47 | 0.004 | 3.14 | 0.012 | NM_000689 |
| 205094_at | PEX12 | peroxisomal biogenesis factor 12 | -2.08 | 0.006 | -1.98 | 0.000 | NM_000286 |
| 219138_at | RPL14 | ribosomal protein L14 | -2.46 | 0.005 | -2.53 | 0.014 | NM_003973 |
| 216563_at | ANKRD12 | Ribosomal protein L18a homologue | -2.08 | 0.013 | nc | 0.013 | X80821 |
| 209785_s_at | PLA2G4C | phospholipase A2, group IVC (cytosolic, calcium-independent) | -2.81 | 0.000 | -3.02 | 0.010 | AF065214 |
| 201473_at | JUNB | jun B proto-oncogene | 5.26 | 0.010 | 5.29 | 0.010 | NM_002229 |
| 205469_s_at | IRF5 | interferon regulatory factor 5 | 3.27 | 0.001 | nc | 0.007 | NM_002200 |
| 207018_s_at | RAB27B | RAB27B, member RAS oncogene family | -2.37 | 0.004 | -2.58 | 0.006 | NM_004163 |
| 201163_s_at | IGFBP7 | insulin-like growth factor binding protein 7 | -3.09 | 0.001 | -3.19 | 0.000 | NM_001553 |
| 219630_at | PDZK1IP1 | PDZK1 interacting protein 1 | -6.60 | 0.003 | -6.74 | 0.008 | NM_005764 |

a: before IVIG treatment

b: after IVIG treatment

nc: not significantly changed
