# Supplementary material for: Gene Expression Profiling in Peripheral Blood Mononuclear Cells of Patients with Common Variable Immunodeficiency: Modulation of Adaptive Immune Response following Intravenous Immunoglobulin Therapy
Source: PLoS One. 2014 May 15;9(5):e97571. doi: 10.1371/journal.pone.0097571 (PMC4022614; doi:10.1371/journal.pone.0097571)
Supplement: Table S2 — Annotated genes differentially expressed in CVID only after IVIG treatment. (DOC) [file pone.0097571.s002.doc]

| **Table S2. Annotated genes differentially expressed in CVID only after IVIG treatment.** | | | | |  |
| --- | --- | --- | --- | --- | --- |
| **Probe Set ID** | **FC post-treatment Vs healthy control** | ***p* value post-treatment Vs healthy control** | **Gene Symbol** | **Gene Title** | **Accession number** |
| 219859_at | 2.07 | 0.010 | CLEC4E | C-type lectin domain family 4, member E | NM_014358 |
| 209774_x_at | 12.56 | 0.007 | CXCL2 | chemokine (C-X-C motif) ligand 2 | M57731 |
| 201694_s_at | 40.53 | 0.000 | EGR1 | early growth response 1 | NM_001964 |
| 205249_at | 24.25 | 0.006 | EGR2 | early growth response 2 | NM_000399 |
| 211305_x_at | 2.08 | 0.008 | FCAR | Fc fragment of IgA, receptor for | U56236 |
| 202768_at | 25.01 | 0.011 | FOSB | FBJ murine osteosarcoma viral oncogene homolog B | NM_006732 |
| 221170_at | 2.30 | 0.007 | HRH4 | histamine receptor H4 | AF312230 |
| 220266_s_at | 3.40 | 0.004 | KLF4 | Kruppel-like factor 4 (gut) | AF105036 |
| 220528_at | 3.36 | 0.004 | VNN3 | vanin 3 | NM_018399 |
| 209993_at | -3.25 | 0.006 | ABCB1 | ATP-binding cassette, sub-family B (MDR/TAP), member 1 | AF016535 |
| 219521_at | -2.29 | 0.007 | B3GAT1 | beta-1,3-glucuronyltransferase 1 (glucuronosyltransferase P) | NM_018644 |
| 207315_at | -3.08 | 0.011 | CD226 | CD226 molecule | NM_006566 |
| 216836_s_at | -2.62 | 0.005 | ERBB2 | v-erb-b2 erythroblastic leukemia viral oncogene homolog 2 | X03363 |
| 218689_at | -2.34 | 0.003 | FANCF | Fanconi anemia, complementation group F | NM_022725 |
| 204007_at | -2.22 | 0.006 | FCGR3B | Fc fragment of IgG, low affinity IIIb, receptor (CD16b) | J04162 |
| 219028_at | -2.13 | 0.002 | HIPK2 | homeodomain interacting protein kinase 2 | NM_022740 |
| 203596_s_at | -2.15 | 0.007 | IFIT5 | interferon-induced protein with tetratricopeptide repeats 5 | NM_012420 |
| 209828_s_at | -2.34 | 0.002 | IL16 | interleukin 16 (lymphocyte chemoattractant factor) | NM_004513 |
| 211397_x_at | -2.21 | 0.006 | KIR2DL2 | killer cell immunoglobulin-like receptor, two domains, long cytoplasmic tail, 2 | L76669 |
| 211532_x_at | -2.46 | 0.013 | KIR2DS1 | killer cell immunoglobulin-like receptor, two domains, short cytoplasmic tail, 1 | L76668 |
| 220646_s_at | -3.61 | 0.008 | KLRF1 | killer cell lectin-like receptor subfamily F, member 1 | NM_016523 |
| 207734_at | -2.24 | 0.005 | LAX1 | lymphocyte transmembrane adaptor 1 | NM_017773 |
| 203823_at | -2.30 | 0.007 | RGS3 | regulator of G-protein signaling 3 | NM_021106 |
| 206118_at | -2.06 | 0.002 | STAT4 | signal transducer and activator of transcription 4 | NM_003151 |
| 204254_s_at | -2.04 | 0.014 | VDR | vitamin D (1,25- dihydroxyvitamin D3) receptor | NM_000376 |
| 205844_at | -3.03 | 0.007 | VNN1 | vanin 1 | NM_004666 |
| 206142_at | -2.35 | 0.000 | ZNF135 | zinc finger protein 135 | NM_003436 |
| 210757_x_at | -2.17 | 0.003 | DAB2 | disabled homolog 2, mitogen-responsive phosphoprotein | AF188298 |
| 205222_at | -2.24 | 0.012 | EHHADH | enoyl-Coenzyme A, hydratase/3-hydroxyacyl Coenzyme A dehydrogenase | NM_001966 |
| 203925_at | -2.04 | 0.005 | GCLM | glutamate-cysteine ligase, modifier subunit | NM_002061 |
| 202336_s_at | -2.06 | 0.015 | PAM | peptidylglycine alpha-amidating monooxygenase | NM_000919 |
| 202273_at | -2.10 | 0.009 | PDGFRB | platelet-derived growth factor receptor, beta polypeptide | NM_002609 |
| 219048_at | -2.01 | 0.005 | PIGN | phosphatidylinositol glycan anchor biosynthesis, class N | NM_012327 |
| 210407_at | -2.07 | 0.002 | PPM1A | protein phosphatase 1A (formerly 2C), magnesium-dependent, alpha isoform | AF070670 |
| 209146_at | -2.01 | 0.006 | SC4MOL | sterol-C4-methyl oxidase-like | U93162 |
|  |  |  |  |  |  |
